# Lessons learned from early implementation of the Growing Expertise in E-health Knowledge and Skills (GEEKS) program in Nigeria, 2019 – 2021

**DOI:** 10.11604/pamj.2023.46.81.38588

**Published:** 2023-11-09

**Authors:** Audrey Rachlin, Oluwasegun Joel Adegoke, Ester Sikare, Olorunsogo Bidemi Adeoye, Edward Dagoe, Asekun Adeyelu, Herman Tolentino, Jan MacGregor, Samuel Obasi, Gabriel Adah, Abdullahi Bulama Garba, Angela Ukpojo Abah, Josiah Friday, Ferdinand Oyiri, Angela Montesanti Porter, Lois Olajide, Idongesit Wilson, Ramatu Usman, Nnamdi Usifoh, Olasoji Fasogbon, Richard Franka, Margherita Ghiselli, Patrick Nguku, Ndadilnasiya Waziri, Eugene Lam, Omotayo Bolu

**Affiliations:** 1 Global Immunization Division, Centers for Disease Control and Prevention, Atlanta, Georgia, United States of America,; 2 National Stop Transmission of Polio (NSTOP) Program, African Field Epidemiology Network (AFENET), Abuja, Nigeria,; 3 Division of Global HIV and Tuberculosis, Centers for Disease Control and Prevention, Atlanta, Georgia, United States of America,; 4 Peraton Inc, Herndon, Virginia, United States of America,; 5 Department of Planning, Research and Statistics, National Primary Health Care Development Agency (PRS-NPHCDA), Abuja, Nigeria,; 6 The Nigeria Centre for Disease Control (NCDC), Abuja, Nigeria

**Keywords:** Capacity building, public health informatics, district health Information system, expanded program on immunization, routine immunization, Nigeria, E-health, vaccine preventable disease surveillance

## Abstract

**Introduction:**

the Growing Expertise in E-health Knowledge and Skills (GEEKS) program is an applied apprenticeship program that aims to improve informatics capacity at various levels of the national health system and create a sustainable informatics workforce. Nigeria adapted the GEEKS model in 2019 as a mechanism to strengthen data quality and use of routine immunization (RI) and vaccine-preventable disease (VPD) surveillance data among Expanded Programme on Immunization (EPI) staff. Since the start of the GEEKS-EPI program, there has not been a formal assessment conducted to measure the extent to which GEEKS-EPI has been able to build local informatics workforce capacity and strengthen RI and VPD surveillance (VPDS) data quality and use in Nigeria.

**Methods:**

we conducted a qualitative assessment to inform the extent to which GEEKS-EPI has been able to build informatics skillsets to enhance local workforce capacity, foster collaboration across government agencies, and create a sustainable informatics workforce in Nigeria. In-Depth Interviews (IDIs) and Focus Group Discussions (FGDs) were held with GEEKS-EPI supervisors, mentors, and mentees from previous GEEKS-EPI cohorts.

**Results:**

while there were challenges reported during early implementation of the GEEKS-EPI program in Nigeria, particularly early on in the COVID-19 pandemic, participants and supervisors reported that the fellowship provided a framework for building a sustainable RI and VPDS informatics workforce through regular mentorship, peer-to-peer exchanges and Subject Matter Expert (SME)-led trainings.

**Conclusion:**

lessons learned from early implementation of GEEKS-EPI in Nigeria will help to inform its implementation in other countries, where strengthened national RI and VPDS informatics capacity is the primary objective.

## Introduction

Public health informatics, or the systematic application of information, computer science and technology to public health practice, research, and learning, is essential for effective health monitoring and surveillance, ensuring timely access to accurate data [1]. A strong network of informatics professionals contributes to long-term sustainability of health programs, enabling programs to respond to local and regional contexts [2] and advancing overarching goals of the WHO Immunization Agenda 2030 [3]. The Growing Expertise in E-health Knowledge and Skills (GEEKS) program is an applied training program, where fellows working in public health undergo a competency- and project-based learning approach to develop and maintain informatics capacity at various levels of the national health system. The GEEKS program was developed by the US Centers for Disease Control and Prevention (CDC), Division of Global HIV and TB (DGHT) at the Center for Global Health, and previously implemented in Uganda, Ethiopia, Zambia, Thailand and Nigeria, primarily focused on building informatics capacity of Ministry of Health (MoH) staff initially to support HIV/TB and related programs and expanded to immunization programs through the Global Immunization Division [4].

The Federal Ministry of Health (FMoH) in Nigeria adopted District Health Information Software 2 (DHIS2) in 2010 as a platform for electronic reporting of health data to strengthen the National Health Management Information System. In 2017 the routine immunization (RI) modular dashboard was introduced on the DHIS2 platform to report and monitor indicators of RI performance across all states in Nigeria [5]. The African Field Epidemiology Network (AFENET) and its National Stop Transmission of Polio (NSTOP) program were responsible for supporting the Department of Planning, Research and Statistics of Nigeria´s National Primary Health Care Development Agency (PRS-NPHCDA) in its implementation of the RI module and providing technical support for RI data analysis and use alongside other implementing partners [5,6]. To address the need to build informatics capacity in the Expanded Programme on Immunization (EPI) and transfer daily operations of the DHIS2 to the Department of Planning, Research and Statistics of Nigeria´s National Primary Health Care Development Agency (PRS-NPHCDA), Nigeria adapted the GEEKS model in 2019 as a mechanism to strengthen data quality and use of routine immunization (RI) and vaccine-preventable disease (VPD) surveillance data among EPI staff and reduce reliance on partners for data management and analytic support [6]. Government professionals need to be equipped with relevant skills to manage robust health information systems and improve data quality and use to promote the health and well-being of all Nigerians. The objectives: since the start of the Nigeria GEEKS-EPI program in 2019, there has not been a formal assessment conducted to inform how GEEKS-EPI has been able to build local informatics workforce capacity and strengthen RI and VPD surveillance (VPDS) health information system data quality and use. We conducted a qualitative assessment among key stakeholders to obtain various perspectives on whether the GEEKS model was able to strengthen EPI informatics capacity and improve RI and VPDS data quality and use in Nigeria.

## Methods

**Figure 1:**
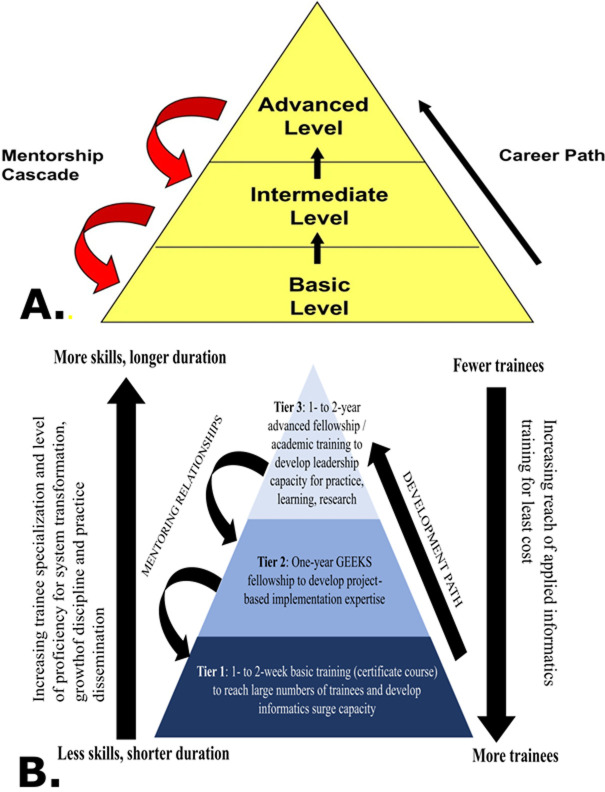
A) FETP training pyramid model [4]; B) GEEKS-EPI structural framework and 3-tiered model adapted from the FETP

**Program description**: GEEKS-EPI is an applied training program for public health officers working in programs that require informatics support. Fellows are selected based on skillset and relevant job descriptions in the Nigeria EPI and include a mix of agency partners: PRS-NPHCDA, National Emergency Routine Immunization Coordination Centre- National Primary Health Care Development Agency (NPHCDA-NERRIC), AFENET/NSTOP, Nigeria Centre for Disease Control (NCDC), and FMoH. GEEKS-EPI aims to develop workforce capacity to improve and strengthen DHIS2 RI and VPDS health information system data quality and use through competency-based trainings as well as on-the-job training complemented by routine mentorship by Subject Matter Experts (SMEs). GEEKS utilizes a three-tiered, project-based, experiential learning approach patterned after the Field Epidemiology Training Program (FETP) developed by the US CDC [4,7] (Figure 1 A). All mentees receive a general foundation in public health informatics through a combination of lectures, group work, and case-based learning (Tier 1), with a smaller subset of mentees advancing to one-year (Tier 2) and two-year (Tier 3) fellowships consisting of mentored projects (Figure 1 B). Two GEEKS-EPI cohorts have completed Tiers 1 and 2 of the program since its introduction through participation in seven targeted projects. All projects centered around improving RI and VPDS health information system data quality and use by the Ministry of Health (MoH) staff and are further described in Table 1. No cohorts have yet to participate in Tier 3 of the GEEKS training program.

**Table 1:** GEEKS-EPI mentored projects completed by cohort one and cohort two fellows and participating agencies involved with each project

Cohort	Timeline	GEEKS Projects and Focus Area	Agencies/Participants	No. of Participants
Cohort One	April 2019-March 2020	Strengthen RI data analysis and its sharing and use for decision-making at all levels of the NPHCDA system	NPHCDA-PRS/AFENET	4
		Monitor the implementation of the DHIS2 RI Module in all States	NPHCDA-PRS/AFENET	3
		Enhance RI data accuracy and completeness at all levels	NPHCDA-PRS/AFENET	2
		Manage and routinely update all back-end programming for DHIS2	NPHCDA-PRS/AFENET	2
Cohort Two	November 2020 – October 2021	Triangulate and visualize routine immunization and VPD surveillance aggregate and case-based data	NPHCDA-PRS/NCDC/AFENET	5
		Enhance RI and VPDS data accuracy and completeness at all levels	NPHCDA-PRS/NPHCDA-NERICC/FMoH/AFENET	4
		Strengthen RI and VPDS data analytics and use through systematic procedures to compare the data from DHIS2, Short Message Service (SMS) and external surveys	NPHCDA-PRS/NPHCDA-NERICC/FMoH/AFENET	4

**Roles of GEEKS-EPI Nigeria participants**: organizations participating in the GEEKS-EPI fellowship nominated mentees and mentors to participate in each cohort. Mentees participated in GEEKS trainings and assignments and actively partook in assigned GEEKS projects under the guidance of mentors. Mentors were tasked with facilitating the planning and implementation of GEEKS projects, supporting mentees in the development of workplans, participating in monthly/quarterly review meetings and implementing recommendations provided by GEEKS supervisors following monthly and quarterly review meetings. Both mentors and mentees were considered GEEKS-EPI fellows. Supervisors provided oversight and leadership to mentors and mentees within their organization and supported the overall implementation of GEEKS-EPI in Nigeria.

**Study design**: the assessment aimed to describe how implementation of the GEEKS-EPI program has enhanced the local informatics workforce capacity in Nigeria related to improved RI and/or VPDS informatics skillsets, how the skills gained have contributed to RI and/or VDPS activities within the EPI, and in what ways the program has affected roles and responsibilities of the local informatics workforce with regards to RI and/or VPDS activities. It also sought to describe how implementation of GEEKS-EPI has affected government cross-agency collaboration, especially in terms of changes in data sharing and access across various agency units. Lastly, it aimed to describe how resources and investments made in the GEEKS-EPI program have been sustained in terms of the utilization and transfer of informatics skillsets gained and integration of activities into the routine local informatics workforce in Nigeria.

**Setting**: the qualitative assessment took place in Abuja, Nigeria in March of 2022 and involved the collection of opinions around how the GEEKS-EPI program has strengthened informatics capacity and supported ongoing activities to improve RI and VPDS data quality and use in-country.

**Participants**: we conducted four IDIs with GEEKS-EPI supervisors and key partners from NCDC, FMoH, and PRS-NPHCDA. We also conducted four FGDs with mentors and mentees from each of the two GEEKS cohorts, consisting of four mentees and five mentors from cohort one, and seven mentees and three mentors from cohort two. Only mentors and mentees who participated in the program for at least 12 months were included in the FGDs. Tier one participants, who only participated in the program for one-to-two week basic trainings, were not included in the FGDs.

**Data analysis**: notes from IDIs and FGDs were de-identified using a data summary worksheet. Audio recordings were transcribed and kept in designated password-protected folders along with other documents including notes and data summary worksheets. A simple manual thematic content analysis was conducted in Microsoft Excel using a list of themes that were identified and operationally defined a priori to align with the study's objectives (Supplementary Data 1). The analysis outcomes examined whether respondents believed the GEEKS-EPI program was able to: 1) build informatics skillsets to enhance local RI and VPDS workforce capacity, 2) foster collaboration across government agencies, and 3) sustain informatics skillsets gained through the integration of activities into the routine local informatics workforce in Nigeria.

**Informed consent**: all participants provided written and verbal consent prior to starting interviews. Participants were de-identified and paper-based logs were kept in a secure and locked location separate from other study documents. To limit potential bias, only external interviewers who played no role in GEEKS-EPI implementation were selected to conduct IDIs and FGDs.

**Funding**: the project received funding from the US Centers for Disease Control and Prevention (CDC).

**Ethical approval**: this activity was reviewed by CDC and was conducted consistent with applicable federal law and CDC policy (45 C.F.R. part 46.102(l)(2), 21 C.F.R. part 56; 42 U.S.C. 145 Sect. 241(d); 5 U.S.C. Sect. 552a; 44 U.S.C. Sect. 3501 et seq). This project was additionally reviewed and approved by the National Emergency Routine Immunization Coordination Centre (NERICC).

## Results

### Successes of GEEKS-EPI Program implementation

**Capacity building**: mentees and mentors from both cohorts recognized the value of the GEEKS-EPI program for improving RI and VPDS informatics skillsets through various capacity building and training activities conducted. Activities covered a wide variety of topics, including foundational informatics trainings, data triangulation, DHIS2 use and back-end system administration, geographic information system (GIS) applications such as QGIS, R Studio, scientific writing, and the use of PowerPoint for developing effective public health presentations. All mentees reported that the hands-on experience gained through participation in GEEKS projects as well as routine guidance and mentorship received were key to further developing and refining the skillsets acquired during the training sessions.

GEEKS-EPI mentees identified three major areas of skillsets acquired: data analysis, data management, and soft skills such as stakeholder engagement and presentation skills. All mentees reported they had improved health informatics skillsets related to the analysis of state-level RI performance indicators, data triangulation and visualization, and use of analytic software including R Studio, QGIS and Microsoft Excel. Data management skillsets requiring importing and exporting data, creation of scorecards, and DHIS2 server administration and back-end administration were also reportedly improved by all mentees. Additionally, four mentees said they were able to improve soft skills including providing more effective feedback and recommendations to officers at state and Local Government Areas (LGA) levels, designing and delivering presentations, and writing scientific reports, abstracts, and manuscripts. Three cohort one fellows (comprising both mentees and mentors) said that were able to attend the Health Informatics in Africa (HELINA) Conference held in Gabon, Botswana in 2019 to present on some of the results of their GEEKS-EPI projects.

Fellows reported that the GEEKS-EPI program had enhanced local RI/VPDS informatics capacity in Nigeria through the application of acquired skillsets to routine informatics activities related to RI/VPDS data management, data quality strengthening, data analysis, and data feedback as described in Table 2 [8-11]. There were notable differences reported in the ways cohort one and two fellows described how their roles and responsibilities had changed since implementation of the GEEKS-EPI program. Cohort one fellows, who participated in the fellowship before the COVID-19 pandemic, all reported serving in much of the same roles with additional responsibilities related to data analysis, quality, reviewing and presenting RI data to subnational levels. Cohort two fellows, who participated in the fellowship throughout early stages of COVID-19 pandemic, noted that several of their roles and responsibilities had shifted due to changed demands of the COVID-19 response. Two cohort two mentees reported that GEEKS projects and agency roles were centered around COVID-19 data analysis and data management throughout the fellowship. For example, one described that they had supported the development and maintenance of the Electronic Management of Immunization Data (EMID) app for COVID-19 vaccine data [12].

**Table 2:** application of skills gained during GEEKS-EPI related to RI and/or VDPS activities in Nigeria as reported by fellows

Activity	Application of Skills
**RI/VPDS Data Management**	Supported data management for National Emergency Routine Immunization Coordination Centre (NERICC) and National Emergency Maternal and Child Health Intervention Centre (NEMCHIC)
	Advised state and local data managers on effective organization, storage, and analysis of data
**RI/VPDS Data Quality Strengthening**	Assisted PRS-NPHCDA with monthly data review, which has decreased the degree of incomplete reporting and the disparity between survey data and administrative data
	Served as focal points for data quality improvement planning
**RI/VPDS Data Analysis**	Analyzed state-level RI data to flag issues related to low-performance indicators and provided feedback
	Utilized data triangulation of vaccine administrative and survey data from different sources including Supplementary Immunization Activities (SIAs), Multiple Indicator Cluster Surveys (MICS), National Demographic and Health Survey (NDHS), Lot Quality Assurance Sampling (LQAS)[8-11]
	Developed National COVID-19 Adverse Event Following Immunization (AEFI) dashboard and indicators
	Conducted routine RI and COVID-19 data analysis for respective agencies
**RI/VPDS Data Feedback**	Generated reports from health facility (HF) data using DHIS2 Applied acquired data visualization techniques for presentations
	Delivered presentations on RI/VDPS data to stakeholders at meetings (e.g. NERICC meetings)

**Cross-agency collaboration**: four fellows described notable improvements related to cross-agency data sharing as a result of GEEKS-EPI implementation. Improved collaboration reported across multiple agencies including FMoH, NCDC and NPHCDA had led to an increase in DHIS2 data sharing and access to Surveillance Outbreak Response Management and Analysis System (SORMAS) data. One fellow noted they were able to engage with agencies involved in the data collection process, such as the National Bureau of Statistics, and regularly received interagency requests related to COVID-19 and RI data analysis and data management. This collaboration has reportedly led to easier access to information, facilitating quicker analysis and feedback of data. One supervisor also reported that GEEKS had provided them the opportunity to view and compare systems utilized by other agencies; thereby encouraging them to improve various aspects of their own health information system, such as usability of data and data visualization.

**Sustainability**: mentees and mentors from both cohorts noted that the skills acquired during the GEEKS-EPI fellowship were maintained since they were frequently used in the daily operations of their respective agencies. Two fellows reported that they were now responsible for training and mentoring agency colleagues not formally trained by GEEKS-EPI. For instance, a fellow from PRS-NPHCDA said that they had organized several trainings for colleagues on how to access and analyze DHIS2 data and were often requested to present on what they had learned after GEEKS-EPI trainings to agency colleagues.

Additionally, four mentees described receiving continuous feedback and follow-up from mentors that helped them to stay motivated and sustain the skills learned post-fellowship. Both mentees and mentors reported receiving additional roles and responsibilities from their home agencies as the fellowship progressed, thus allowing them to maintain and further improve the skillsets acquired through the GEEKS-EPI program. One fellow had also participated in post-GEEKS fellowship trainings and continued to build their own capacity through personal learning efforts.

**Key Accomplishments of GEEKS-EPI Nigeria:** one recorded outcome of GEEKS-EPI implementation in Nigeria was the expansion of both RI/VPDS and COVID-19 immunization dashboards developed by fellows as part of their GEEKS-EPI projects. One fellow noted that they had helped to support the development and management of a national RI/VPDS dashboard triangulating SORMAS surveillance data with vaccine administrative and survey data. Another fellow also said they had played a role in the establishment of the national COVID-19 Adverse Event Following Immunization (AEFI) dashboard including key AEFI indicators, while another had helped the development and maintenance of the EMID app for COVID-19 vaccination through regular server management and data analysis.

Additionally, GEEKS-EPI fellows were responsible for improvements observable on the DHIS2 RI dashboard. Six mentees described conducting monthly analyses on data completeness and accuracy to identify performance issues related to vaccination coverage. They noted summarizing these challenges and providing recommendations in monthly reports to provide subnational feedback to respective states. Through this targeted support, improvements were apparent on the DHIS2 RI dashboard, particularly in states reporting more frequent data quality issues. For example, incorrect entry of data related to dose one and two of the hepatitis B vaccine (HepB) on the DHIS2 RI dashboard declined by 20% between April 2019 and October 2019 following feedback provided by GEEKS fellows to subnational levels, indicating that such data should not be entered in the RI dashboard as they are not included in the Nigeria immunization schedule (In Nigeria only HepB pediatric, given at birth, is included in the immunization schedule. HepB first and second dose are not included the national schedule) [13].

**Challenges and Limitations of GEEKS-EPI program implementation**: the GEEKS-EPI program encountered several challenges related to its introduction across cohorts one and two:

**Competing agency priorities**: agency activities often interfered with GEEKS-EPI trainings and projects. Two supervisors and three participants mentioned that without a comprehensive timetable, fellows were not able to schedule time to participate in GEEKS activities and trainings with supervisors in advance. This frequently led to conflicts in scheduling between agency and program activities.

**Suboptimal trainings**: fellows also noted having limited input in their assigned projects and that capacity building trainings were not always optimal. According to three fellows, projects were not always relevant to current agency roles and interests, leading to a general lack of engagement. Five fellows also noted that more in-depth trainings targeted to specific agencies and job roles would have also been beneficial, particularly related to data analysis and visualization (e.g., PowerBI, SPSS, QGIS, and R).

**Logistic challenges**: additionally, both cohorts reported that the lack of resources they were provided made it challenging to participate at certain times. Cohort one mentees did not receive data bundles or laptops until the end of the fellowship, and cohort two did not receive these at any point during the fellowship. Two cohort two mentees noted they had no laptop provided through their respective agencies, making it difficult to participate in virtual trainings and complete activities or assignments.

**Ongoing participation and engagement**: lastly, there were no established mechanisms for fellows to continue engaging with the GEEKS-EPI program following completion, or ongoing opportunities for learning or training provided. Four fellows mentioned this would have promoted ongoing involvement with the program and networking with other fellows long-term. According to many fellows, ongoing engagement and learning opportunities were key to help them further develop newly acquired skillsets and continue taking on additional informatics responsibilities within their agency. One recommendation for this could be for GEEKS-EPI graduates to join the Shiriki community [14], an online platform that prior GEEKS DGHT HIV/TB graduates have joined for the purpose of continuing informatics education and knowledge sharing of intermediate to advanced topics.

**COVID-19-specific challenges**: there were challenges specific to GEEKS-EPI cohort two, who participated in the fellowship throughout the COVID-19 pandemic [15]. Across all agencies, fellows reported that pandemic activities and fieldwork took precedence over GEEKS projects and trainings, and they often had a difficult time balancing multiple priorities. With nearly all trainings and mentoring sessions held virtually, fellows also reported that those who didn´t have laptops provided through their agencies struggled to join calls or complete assignments. Additionally, all cohort two fellows said they were not able to attend GEEKS-EPI trainings in-person or join conferences, seminars, or presentations held outside of their agency´s base in Abuja, Nigeria. This had reportedly led to issues around distraction, with agency or family activities often taking precedence around trainings. Four fellows also mentioned there were limited opportunities for networking with other GEEKS-EPI fellows and health informatic professionals during this time, and two mentees reported that these challenges had collectively led to a general lack of motivation and engagement in the program. These lessons are relevant for ongoing implementation of the program in Nigeria and potential future implementation in other countries considering GEEKS-EPI as a mechanism to improve informatics RI/VPDS informatics capacity.

## Discussion

**Lessons learned and recommendations for future GEEKS-EPI programs**: although there were challenges reported during implementation of the GEEKS-EPI program in Nigeria, particularly at the start of the COVID-19 pandemic, all fellows and supervisors collectively agreed that the program provided a framework for building RI and VPDS informatics capacity through regular mentorship, peer-to-peer exchanges and SME-led trainings. To further strengthen the GEEKS-EPI program in Nigeria, and in other countries considering implementing similar programs, we have provided several recommendations:

**Projects and trainings**: program onboarding and key trainings should be done in-person and outside of the city where fellows are based. This will help to limit issues with distraction and competing agency or external priorities. As part of this, an advanced schedule of all trainings could be provided to fellows, so they are able to request time off in-advance with supervisors, limiting the number of unforeseen competing agency activities. Additionally, supervisors could sign a memorandum of understanding (MOU) stating that fellows are allowed time to attend GEEKS training. Engaging agencies early to understand gaps and interested personnel could lead to identification of projects that address specific needs and targeted recruitment of staff to participate as mentees. Likewise, fellows could also be encouraged to have more input on project topics to ensure engagement and relevance to their agency´s work. Integration of more advanced, in-depth trainings on topics that are most relevant to fellows as part of their agency roles might also be considered, including those related to data analysis and visualization (PowerBI, SPSS, QGIS, R, etc.), as well as scientific writing trainings. External consultants with additional expertise in these areas could deliver trainings where funds allow.

**Opportunities for Fellows**: fieldwork opportunities at the subnational level are central to help fellows contextualize the realities and challenges associated with data collection and input. These opportunities should be prioritized where possible. Additional opportunities for fellows to participate in conferences and seminars, both local and international are also critical to help fellows broaden their network, promote deeper understanding, and reinforce existing best practices.

**Following Completion of the GEEKS-EPI Program**: a quality improvement plan could be developed incorporating indicators for routine program monitoring and plans for ongoing training of mentors and supervisors, which will help to facilitate continuous strengthening of mentorship and supervision quality. A post-fellowship assessment, both practical and skills-based, could also be conducted to record what skills have been gained and assess any gaps remaining. Continued networking and engagement in GEEKS-EPI following completion of the fellowship should also be encouraged. This could be done through the development of a GEEKS-EPI alumni portal or through ongoing opportunities for fellows to work as mentors as their skillsets become more advanced, which will also help previous trainees to remain engaged in the program while continuing to build skills internally. Finally, instances for fellows to continue engaging in further trainings and study once they have completed the program are important to ensure continued strengthening of newly acquired skillsets and ongoing intra-agency capacity building.

**Assessment limitations**: our study had several limitations. First, our qualitative analysis provided only a partial interpretation of how GEEKS-EPI has been able to build local health informatics workforce capacity and strengthen RI and VPD surveillance (VPDS) data quality and use in Nigeria. Future iterations of this study could involve quantitative metric indicators and additional desk review to further examine the extent to which the fellowship has improved RI and VPDS health informatics performance. Second, we used a simple manual thematic content analysis to simplify interpretation. Although we could have used more complex analyses, given the small number of IDIs and FGDs we were able to conduct, these techniques would have been difficult to interpret. Third, not all fellows and supervisors who participated in the GEEKS-EPI program were available to participate in our study and only those who participated for greater than 12 months were included in the assessment, therefore it is possible additional interpretations and viewpoints were missed. In view of all these limitations, our study should be interpreted as a starting point for any robust future assessment of similar programs.

## Conclusion

The GEEKS-EPI fellowship provides a framework for strengthening national RI and VPD surveillance data quality and use by fostering local informatics workforce capacity through routine mentorship, peer-to-peer exchanges and trainings. Lessons learned from implementation of GEEKS-EPI in Nigeria will help to inform its implementation in other countries, where strengthened RI and VPDS informatics capacity is the primary objective**.**
